# On the Use of Temperature Measurements as a Process Analytical Technology (PAT) for the Monitoring of a Pharmaceutical Freeze-Drying Process

**DOI:** 10.3390/pharmaceutics15030861

**Published:** 2023-03-07

**Authors:** Alberto Vallan, Davide Fissore, Roberto Pisano, Antonello A. Barresi

**Affiliations:** 1 Department of Electronics and Telecommunications, Politecnico di Torino, Corso Duca Degli Abruzzi 24, 10129 Torino, Italy; 2 Department of Applied Science and Technology, Politecnico di Torino, Corso Duca Degli Abruzzi 24, 10129 Torino, Italy

**Keywords:** freeze-drying, process monitoring, temperature measurement, temperature sensors, thermocouple, pharmaceuticals

## Abstract

The measurement of product temperature is one of the methods that can be adopted, especially in the pharmaceutical industry, to monitor the freeze-drying process and to obtain the values of the process parameters required by mathematical models useful for in-line (or off-line) optimization. Either a contact or a contactless device and a simple algorithm based on a mathematical model of the process can be employed to obtain a PAT tool. This work deeply investigated the use of direct temperature measurement for process monitoring to determine not only the product temperature, but also the end of primary drying and the process parameters (heat and mass transfer coefficients), as well as evaluating the degree of uncertainty of the obtained results. Experiments were carried out with thin thermocouples in a lab-scale freeze-dryer using two different model products, sucrose and PVP solutions; they are representative of two types of commonly freeze-dried products, namely those whose structures are strongly nonuniform in the axial direction, showing a variable pore size with the cake depth and a crust (leading to a strongly nonlinear cake resistance), as well as those whose structures are uniform, with an open structure and, consequently, a cake resistance varying linearly with thickness. The results confirm that the model parameters in both cases can be estimated with an uncertainty that is in agreement with that obtained with other more invasive and expensive sensors. Finally, the strengths and weaknesses of the proposed approach coupled with the use of thermocouples was discussed, comparing with a case using a contactless device (infrared camera).

## 1. Introduction

Freeze-drying is widely employed to increase the shelf life of pharmaceutical products, especially when liquid formulations must be processed in a vial or tray, but recently other containers, such as syringe cartridges or blisters, have also been considered. Water is removed via sublimation at low temperatures and pressures, and thus, it is generally considered the drying process of choice for thermolabile products, even if the freezing step and the solute concentration that occurs because of the formation of the ice crystals may also be dangerous, especially for biomolecules. Ice sublimation is an endothermal phenomenon; thus, heat must be supplied during primary drying at the maximum possible rate to speed up the process. The shelf temperature can be relatively high, as the heat transfer coefficient between the shelf and the bottom of the container is low as an effect of the very low pressure, but the product temperature remains low until there is ice that sublimates if the mass transfer resistance of the dried cake is sufficiently small to allow the flux of the vapor formed. If the driving force is not sufficient to allow this flux, heat accumulates in the product, increasing its temperature; in fact, the higher temperature at the frozen–dry layer interface increases the vapor tension of ice and thus, the driving force and flux [1,2,3,4].

Thus, during primary drying, the product temperature must be maintained at the highest possible value below a threshold to preserve the final quality: both activity and appearance may be affected, and as far as what concerns the stability, not only must the product yield immediately after the completion of freeze-drying be taken into account, but also the long-term storage stability under specified conditions [5,6,7].The threshold temperature mentioned above may be related to thermal degradation of the product: the effect of temperature on stability has been discussed by Chen et al. [8] and by Wang and Pikal [9], who highlighted the importance of molecular motions on protein stability in the amorphous solid; since molecular mobility is significantly impacted by thermal history, variation in lyophilization processing conditions could produce big differences in product stability. Trespassing the threshold temperature during drying may cause the loss of the structure as a consequence of collapse: for amorphous products, the reference is generally the glass transition temperature of the “dried” product in contact with the frozen one, which has the highest residual moisture content [10]; collapsed formulations are often unacceptable by requested standards and show generally higher moisture content and longer reconstitution time. Anyway, Depaz et al. [11] showed that at higher protein concentrations, pharmaceutically acceptable cakes may be obtained for formulations freeze-dried above *T_g_*′ but below the collapse temperature. A non-ideal cake appearance is often rejected by quality protocols, even if it has been shown that it does not always impact product quality [12]. Actually, the majority of the investigations have found little or no decrease in product quality for formulations freeze-dried above the collapse temperature, while in a few studies some decrease in long-term stability was observed [13]; this is very interesting because the slight but controlled increase in process temperature that might be allowed would lead to a significant increase in productivity. For crystalline substances, the limit is generally the eutectic temperature; when trespassing it, some liquid forms, causing boiling for the low pressure and a final foamy appearance.

In the secondary drying step, shelf temperature and thus product temperature is increased to favor the desorption of the bound water which did not freeze, but remained adsorbed to the product. As the collapse temperature decreases with the residual moisture, higher temperatures are generally set in this step to accelerate water desorption; a proper temperature setting, as well as the correct identification of the start of secondary drying (with the corresponding temperature increase) above all, is important to reduce overall drying time and avoid collapse.

During the freeze-drying process, therefore, it may be important:

- to monitor product temperature, as it must remain below the threshold value to preserve final product quality. Usually, both product temperature at the bottom of the vial (*T_B_*) and that at the interface of sublimation (*T_i_*) should be known.
- to identify the occurrence of the ending point of the primary drying stage, corresponding to the time instant when no more ice is present or the thickness of the frozen layer (*L_froze_*_n_) approaches zero, if a flat and planar interface is assumed.
- to estimate the value of the parameters of a mathematical model of the process. The heat transfer coefficient (*K_v_*) and the resistance to mass transfer (*R_p_*), which allow us to describe, respectively, the heat transfer to the product and the mass transfer from the product to the drying chamber, are generally selected. They can be used to predict the effect of the operating conditions and thus to optimize the process, minimizing the duration of the drying stage [14,15].

This may be performed by developing a suitable process analytical technology (PAT), which would allow us to monitor and eventually control the process in-line, thus assuring that the desired quality is obtained in the final product. This approach is totally different from the traditional quality-by-testing approach, and it was strongly encouraged by “Guidance for Industry PAT”, which was issued by the FDA in September 2004 with the goal of producing effective, safe, and affordable medicines [16]. An extensive review of the systems that have been proposed to achieve these goals can be found in the literature. Barresi and coworkers [14,17] catalogued the various techniques proposed, considering the size of the sample (monitoring single vials, a group of vials, or the whole batch). Alternatively, they could be grouped on the basis of monitored variables, which could be a specific physical property (e.g., temperature or moisture), a flux (heat or sublimation flux) [18,19], or the identification of the ending point of the primary drying stage [19,20,21]. It must be evidenced that measuring devices should be able to supply the value of all the variables of interest—here, temperature and residual moisture (that is, the full state of the system)—to be applicable in a predictive control system.

Measuring the product temperature is probably the most used and powerful means of monitoring the process. Combined with the use of a process model, it can allow us to reach all the goals listed above, including the identification of the end of primary drying and the model parameters. It must be evidenced that the different approaches may be based on either the direct or the indirect (inferential) measurement of the product temperature; in the second case, the direct monitored variable may be the heat flux or the sublimation flow.

The pressure rise test is the base of a first group of indirect systems: it is a standard step-identification approach using the measurement of the chamber pressure, which increases during the test as a function of the sublimation rate; it provides “mean” values of the desired variables and can simultaneously estimate the process parameters [22,23,24,25,26]. The “mean” product temperature may also be estimated using the direct measurement of the sublimation flow using the TDLAS, but in this case, the *K_v_* parameter must be known [27].

Unfortunately, the batch is generally nonhomogeneous [28], and to manage this heterogeneity, it is important to have the distributed values of the variables of interest [29]. The use of heat flux sensors, which was recently proposed, may allow us to obtain local values, but the accuracy and the practical use of this type of device still has some serious limitations [30]. In this respect, process monitoring via direct temperature measurement is more powerful because in principle, the most important or critical zones can be monitored separately using contact or contactless devices.

Regarding contact systems, resistance temperature detectors (RTDs) and thermocouples are the traditional ones generally used in lab and production apparatus: their advantages and limitations have recently been discussed in detail, also revising best practices, and comparing them to newly proposed devices [17,18]. As concerns the latter, TEMPRIS technology [31] has found interesting applications, especially after the size of the probe was significantly reduced, while optical fiber systems (OFSs) have promising characteristics, but are still a subject of research [32].

The temperature sensor most commonly employed to monitor the process and to map the non-uniformity of the batch is the thermocouple because of its reduced invasiveness and low cost [18,33].When the sensor–product interference is a concern, non-invasive monitoring of the product is possible using thermocouples sputtered on the vial external surface and obtained through low-pressure plasma processes; in this case, it is only required that the cake be in contact with the internal vial body [34,35,36]. An indirect sensing approach that allows for a non-invasive measurement via flexible multi-point sensing probe using thermistors, photosensitive lithography, and chemical etching was also recently proposed [37].

Anyway, because of their low cost, easy handling, and flexibility, standard thermocouples are widely employed in both small- and large-scale freeze-dryers when monitoring the temperature evolution in a large number of vials and in different positions of the batch is required. To this aim, thermocouple measurement devices, also called readers, can be designed using simple conditioning circuits with low power consumption and are able to withstand the severe ambient conditions normally found inside freeze-dryers without significantly impairing the measurement accuracy. Examples of battery-operated measurement devices for thermocouples that also embed a wireless communication link have been developed and tested in real conditions [38,39]. A further advantage of these systems is their flexibility and the reduction in the sensor wires routed inside the vacuum chamber.

Thermocouple measurement devices can be also designed to work outside the drying chamber. This solution has the advantage of simpler and more accurate electronics, but the thermocouple wires must be routed outside the chamber using a vacuum stopper, which is a drawback of this approach. Moreover, further problems arise for the sensor calibration, since the thermoelectric circuit spans from the thermocouple tip to the measurement devices through the stopper, and thus, a full thermometer calibration should also involve the stopper wires.

An infrared camera can be an alternative tool to capture product temperature in a freeze-drying cycle without being in contact with the monitored product. The main drawback is represented by the harsh conditions that are experienced by the sensor in the drying chamber, i.e., a temperature that may reach values as low as −40 °C or −50 °C (in the freezing step) during the cycle, a low pressure, and a gas composition that is about 100% water vapor. For these reasons, Emteborg et al. [40,41] placed the camera outside the drying chamber, on the top of the freeze-dryer, using a germanium window. In addition, also Van Bockstal et al. [42] placed the infrared camera outside the freeze-dryer, although in their case, a continuous freeze-drying process was monitored.

Up to now, the only device that was designed to be placed in the drying chamber and to monitor the (axial) product temperature in the vials used for manufacturing was designed by IMC Service S.r.l. (Italy) and firstly tested by Lietta et al. [43,44,45]. The system encompasses a standard infrared camera and a protective enclosure in a thermally insulating plastic material, transparent to Wi-Fi communications.

This work focuses on thermocouples with the aim of deeply investigating the use of direct temperature measurement for process monitoring, not only to determine the product temperature, but also to determine the end of primary drying and the process parameters that allow process optimization (and, in perspective, real process control). A preliminary analysis of the use of thermocouples for process monitoring was discussed by Fissore et al. [46]. This paper extends their investigation, focusing on the combination of this measure with a model of the process to estimate the desired variables and analyzing the main uncertainty contributions from a metrological point of view. A comparison of the measurement results obtained with different products and methods is eventually provided. At the end, the reliability of the approach for the pharmaceutical field is discussed, evidencing strengths and weaknesses of the proposed method based on thermocouples and comparing them with advantages and limitation of the contactless approach.

## 2. Procedure for Process Monitoring Using Temperature Measurement

### 2.1. Determination of the End of Primary Drying

After the initial temperature increase at the beginning of the primary drying, once stationary conditions are attained, the product temperature remains low and practically constant until the heat supplied is used for ice sublimation. After ice sublimation is completed, the heat supplied is used to increase the product temperature until it reaches the temperature of the heating element. Thus, the measurement of product temperature can be used to estimate the ending point of the primary drying stage, looking at variations in the slope of the temperature evolution.

### 2.2. Identification of Process Parameters

In addition to the ending point, as was highlighted in the Section 1, it is quite often necessary to also estimate the value of the parameters of a mathematical model that is then used for process optimization, aiming to minimize the duration of the drying stage. Several models were proposed in the past to describe the freeze-drying process in vials and trays, but the majority of them are characterized by two parameters, namely *K_v_* and *R_p_*. Velardi and Barresi [47] have analyzed the effect of the various hypotheses and simplifications adopted, discussing the accuracy versus the complexity of various models as a function of the application. The model proposed used only two parameters to be identified, assuming negligible radial gradient of temperature and composition and, thus, a planar interface separating the dried and the frozen product. This was considered a suitable compromise for monitoring purposes; in fact, it was used extensively in the past both for off-line optimization, to calculate the design space of the primary drying stage [48,49], and for in-line optimization, e.g., in the *LyoDriver* algorithm [50].

**Figure 1:**
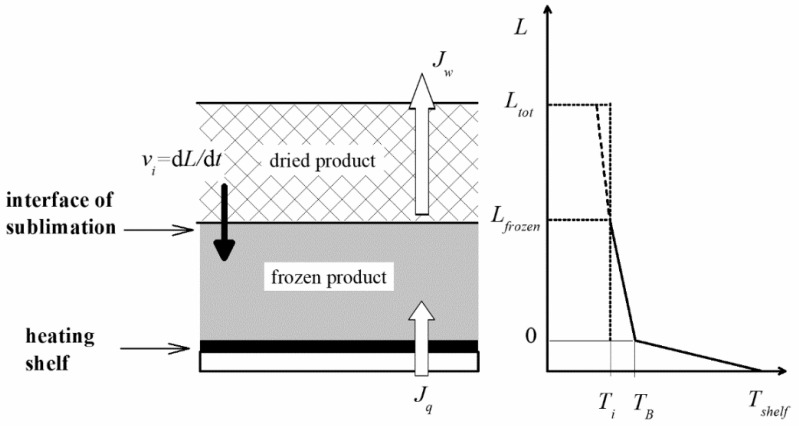
Sketch of the one-dimensional freeze-drying model for primary drying. On the right is an example of the axial temperature profile, evidencing *T_B_*, *T_i_*, and the position of the interface (for completeness, the temperature in the dried part is also shown with a dashed line).

The same model is adopted in this work, considering the simple general case of heat supplied by conduction through the lower shelf, with the frozen–dry interface moving downward as drying goes on; a scheme with an indication of the main variables is shown in Figure 1.

The procedure to be used for obtaining the value of the process parameters from the temperature measurements is the following.

(a) The value of the coefficient *K_v_* can be calculated in a preliminary experiment using the measurement of product temperature. In fact, from the integral energy balance for the frozen product, where *K_v_* can be obtained as the ratio of the total energy supplied to the sample and then used to sublimate the initial mass *m* of ice:
  (1a) $$K_{v}=\frac{m\Delta H_{s}}{A_{v}\int_{0}^{t_{drying}}\left(T_{shelf}-T_{B}\right)dt}$$

The shelf temperature *T_shelf_* might be measured directly using additional thermocouples, taking care to have a good thermal contact; alternatively, it is possible to use the measured value of the technical fluid, and in this case, *K_v_* also includes the resistance of the shelf. The use of Equation (1a) requires estimating the primary drying time (*t_drying_*) with an acceptable accuracy.

Alternatively, a direct measurement of *K_v_* can be obtained using the gravimetric test [15]: at the time *t*_1_ (before the ending of the ice sublimation) when the product is removed from the drying chamber, the vial with residual ice is weighed, and the mass of sublimated ice is calculated via difference. This allows us to calculate *K_v_* without any uncertainty on the duration of the sublimation stage, using Equation (1b):

(1b) $$K_{v}=\frac{\Delta m\Delta H_{s}}{A_{v}\int_{0}^{t_{1}}\left(T_{shelf}-T_{B}\right)dt}$$

where the value of *m* is replaced by the measured weight loss ∆*m* and the drying time is replaced by the duration of the test *t*_1_, but it is necessary to weigh the vials before and at the end of the drying period.

(b) The cake resistance *R_p_* and its variation with *L_dried_* can be calculated using the *K_v_* value calculated in the previous step. The proposed algorithm is the following:
  - The heat flux *J_q_* to the product is calculated with Equation (2),
    (2) $$J_{q}=K_{v}\left(T_{shelf}-T_{B}\right)$$
    using the estimated value of *K_v_* and the measured values of *T_shelf_* and of the temperature of the product at the bottom of the vial (*T_B_*), whose difference represents the driving force.
  - The sublimation flux *J_w_* is then calculated using Equation (3),
    (3) $$J_{q}=\Delta H_{s}J_{w}$$
    the energy balance at the interface of sublimation, considering that in steady-state the heat flow is equivalent to the mass flow times the heat of ice sublimation ∆*H_s_*.
  - The cake resistance can be calculated from the sublimation flux, which, similarly to the heat flux, can be described as the product of a mass transfer coefficient (1/*R_p_*) times a driving force (the difference between water vapor partial pressure at different locations: the interface and the chamber average):
    (4) $$J_{w}=\frac{1}{R_{p}}\left(p_{w,i}-p_{w,c}\right)$$
    During primary drying, it can be assumed that *p_w,c_* is equal to chamber pressure (the fraction of air or inert gas is generally negligible), while the water partial pressure at the interface, *p_w,i_*, can be calculated: the interface temperature is not known, but *T_B_* is measured, and *T_i_* can be estimated by iteratively repeating steps (iii) to (v), neglecting the temperature gradient in the vial as a first attempt, or taking a value (1÷2) °C lower than *T_B_* on the basis of the experience.
  - The sublimation flux in the time interval considered also allows us to estimate the evolution of *L_frozen_* (and thus, as difference to total thickness, of *L_dried_*) using a mass balance to the frozen layer:
    (5) $$v_{i}=\frac{dL_{frozen}}{dt}=-\frac{1}{\rho_{frozen}-\rho_{dried}}J_{w}$$
    and thus, to relate *R_p_* to *L_dried_* for each time interval. Integrating Equation (5), it is also possible to monitor the progress of the drying process in-line.
  - The temperature at the interface can be estimated precisely from the product temperature at the bottom of the container, which is the variable usually measured with thermocouples inserted in vials, considering the heat balance for the frozen product and previous relationships:
    (6) $$T_{B}=T_{shelf}-\frac{1}{K_{v}}{\left(\frac{1}{K_{v}}+\frac{L_{frozen}}{k_{frozen}}\right)}^{-1}\left(T_{shelf}-T_{i}\right)$$
    This, once *L_frozen_* has been calculated from (iv), allows us to calculate *p_w_*_,*i*_ with greater accuracy, and then to calculate *R_p_* using Equation (4).
  - The values of *R_p_* depend on the type of product (and freezing history), but also on the thickness of the dried product, as shown before. To model this dependence, an equation like the following one is usually adopted:
    (7) $$R_{p}=R_{p,0}+\frac{A_{R_{p}}L_{dried}}{1+B_{R_{p}}L_{dried}}$$

The parameters *R_p,_*_0_, $A_{R_{p}}$, and $B_{R_{p}}$ are fitting parameters that can be easily calculated once the curve *R_p_* vs. *L_dried_* has been obtained in step (iv).

## 3. Experimental Set-Up

### 3.1. Experimental Apparatus

A pilot-scale freeze-dryer (LyoBeta 25, Telstar, Spain) with four shelves (total area available for the product: 0.5 m^2^) in a 0.2 m^3^ drying chamber, a vacuum pump, and an external condenser was used for the experimental runs. The service fluid temperature (silicon oil) was monitored using a RTD (Pt100) embedded in the control system of the apparatus. Chamber pressure was monitored with a capacitance gauge (Baratron type 626A, MKS Instruments, Andover, USA) and maintained at the desired value using nitrogen and a controlled leakage system.

The apparatus was equipped with a thermal conductivity gauge (Pirani type PSG-101-S, Inficon, Bad Ragaz, Switzerland). The signal of the Pirani gauge was not used for monitoring the pressure, but for identifying the ending point of the primary drying stage, considering the ratio with the value given by the capacitance gauge [20]. In fact, the thermal conductivity gauge was calibrated into air, and its value was affected by the fraction of water vapor present: only when the primary drying was completed and the atmosphere in the drying chamber contained no vapor did the ratio of the two signals equal one. This corresponded to the end of sublimation in all the vials, but there was some uncertainty in the identification of end of drying, and the onset and inflection point of the curve were also proposed. In fact, it has been evidenced that when the curve starts decreasing, the largest part of the batch has completed primary drying, and the time interval between onset and offset is influenced by the non-uniformity of the batch and by the dynamics of water vapor in the drying chamber [21].

**Figure 2:**
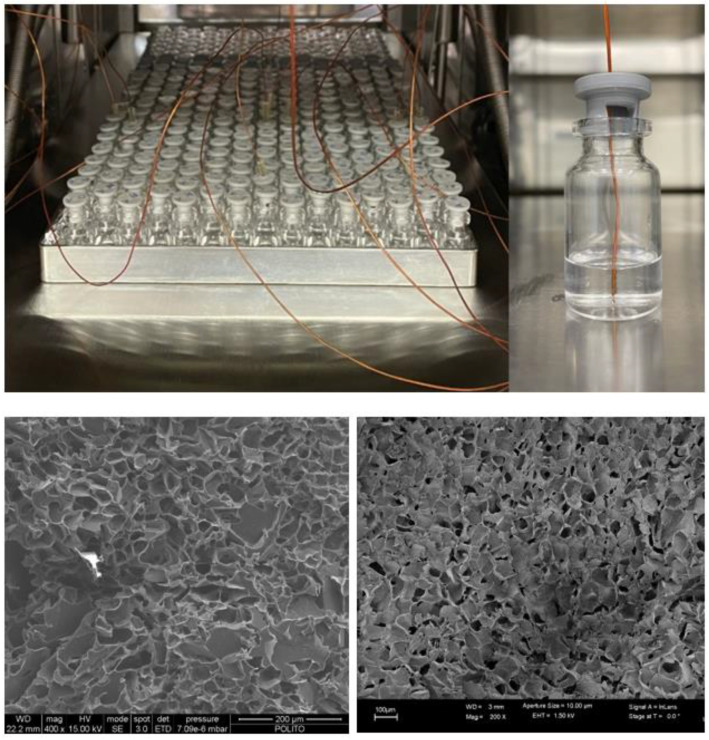
**Upper left**: Example of set up for monitoring the process in a small pilot-scale apparatus, using thin-wire thermocouples. **Upper right**: Detail of the monitored vial, with the thermocouple inserted through the stopper (TC positioners might also be used). Lower graphs, SEM images of the core of freeze-dried samples: (**left**) 5% sucrose solution (metallized sample, bar = 200 μm); (**right**) 5% PVP solution (metallized sample, bar = 100 μm).

Vial temperatures were measured using commercial T-type thermocouples made with 30 AWG single strand wires and exposed tips; the set-up is shown in Figure 2 (upper graphs). The wires were isolated with Kapton, and the overall external diameter of each sensor was about 0.9 mm. Thermocouples were plugged in a custom-made conditioning and acquisition system designed to work inside the vacuum chamber. The overall system was able to measure up to six temperatures with a rate of 1 s. The system was powered and interfaced to an external PC using a wired UART interface. The full measurement chain, from the sensor to the reader, was calibrated against a traceable Pt100 sensor. After the calibration, comparison tests carried out at different temperatures and ambient conditions showed a temperature error below 1 °C.

### 3.2. Case Study

Two different formulations were considered in the study to evaluate the thermocouple-based PAT tool for process monitoring, namely (i) a 5% by weight sucrose (Riedel de Haën) aqueous solution and (ii) a 5% by weight polyvinylpyrrolidone (PVP) (Fluka) aqueous solution. The rationale for this choice was that these two solutions are representative of two types of commonly freeze-dried products, namely those whose structures are strongly nonuniform in the axial direction with the formation of a crust on the top (sucrose, see for example Figure 5 in [51]) and those whose structures are more uniform, with an open structure (PVP). Figure 2 (lower graphs) shows the typical structure of the two products. Experimental results have evidenced the dependence of the average pore size and pore size distribution and their axial distribution both on product type and concentration and on freezing conditions [52,53], while formation of crust seems characteristic of the product, even if the mechanisms have not been completely clarified. The presence of a crust leads to a strongly nonlinear cake resistance, but when the axial distribution is more uniform, the cake resistance varies linearly with thickness. Thus, the selected products should allow us to test the performance of the PAT tool with the two typical shapes of *R_p_* commonly encountered in pharmaceutical applications.

Formulations were prepared using ultra-pure water produced by a Millipore system (Milli-Q RG, Millipore, Billerica, MA). The solutions were then processed in non-GMP conditions into ISO 8362-1 8R glass tubing vials (external diameter = 22 ± 0.2 mm, wall thickness = 1 ± 0.04 mm), pouring 2 mL of liquid in each vial; vials were partially closed using silicon stoppers (West Pharmaceutical Services, Milano, Italy) and loaded onto the shelves of the drying chamber according to a hexagonal array.

Freezing was carried out at −50 °C, with initial cooling decreasing the temperature of the technical fluid at 1 °C/min. The primary drying stage was carried out at 10 Pa and −20 °C (fluid temperature) for the sucrose solution and at −14 °C for the polyvinylpyrrolidone solution. The secondary drying stage was carried out at 20 °C and 2 Pa in both cases.

Thermocouples were inserted in close contact with the bottom in various vials of the batch to measure product temperature *T_B_*, while *T_shelf_* was determined using the RTD inserted in the technical fluid.

## 4. Results and Discussions

### 4.1. Evaluation of PAT Based on Thermocouples

#### 4.1.1. End of Primary Drying

**Figure 3:**
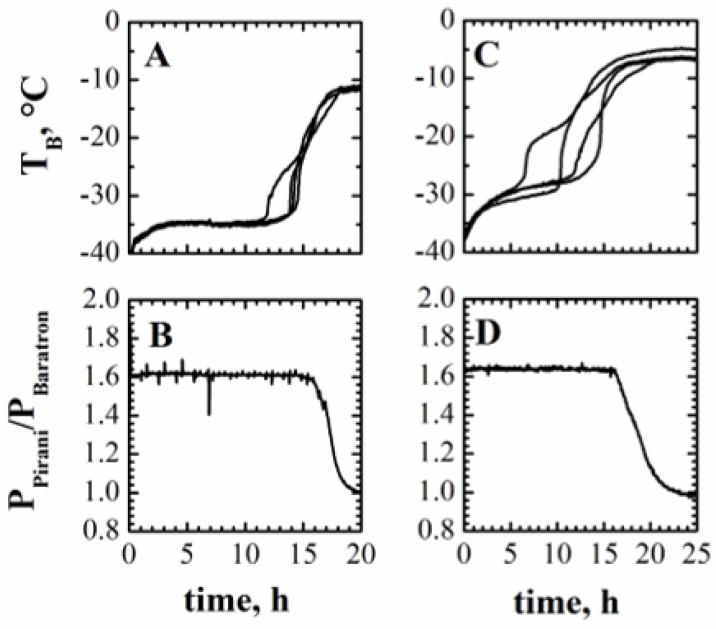
Product temperature measured by thermocouples inserted in 4 different vials (**A**,**C**) and ratio between the signals of the Pirani and Baratron pressure gauges (**B**,**D**) during the freeze-drying of the 5% by weight sucrose solution (**A**,**B**) and of the 5% by weight PVP solution (**C**,**D**).

Figure 3A shows the trend of temperature measurements obtained in four different vials placed in the central part of the batch when the sucrose-based solution was processed. This trend is representative of the product temperature evolution in a freeze-drying process, as discussed in the following section. As discussed in Section 2, the heat supplied during primary drying increases the product temperature up to an almost steady-state value, which should be maintained until the heat is used for ice sublimation. Then, the energy supplied increases the temperature of the dry product, and the temperature measured by the thermocouple rises approaching the temperature of the heating shelf. In practice, a sudden increase in the measured temperature is generally observed well before the end of the primary drying stage, with a large variability from vial to vial. This variability may also be related to incorrect position of the thermocouples. However, it has been pointed out by Bosca et al. [33] that the anticipated sudden temperature increase generally occurs because the front overcomes the thermocouple tip position, but may also be due to irregularity in the ice interface or simply loss of contact between the thermocouple tip and the product. It is worth pointing out that in the presence of a temperature change, the following temperature measurements are no longer representative of the temperature of the frozen product.

On the other hand, an abrupt temperature change may correspond to the end of the sublimation stage in just the monitored vial, which is thus not representative of the whole batch. It must be remarked that the temperature detected in different monitored vials generally is not “exactly” the same; differences can be noted both before and after the “sudden” temperature variation occurs. In fact, in the first part of the primary drying, the heat flux may not be the same for all the vials of the batch, even in the central ones shielded by the edge vials from radiation by the chamber walls. This may be due, among other things, to temperature gradients in the shelf, to the characteristics of the contact area between the vials and the shelf, and/or to the geometric features of the vial bottom. In the case of a well-designed freeze-dryer where the temperature gradients over the shelf are minimized, as well as high-quality vials being used for product processing, with tight tolerance in geometric features, the temperature difference detected in the central vials of the batch may be as low as the accuracy of the temperature probe.

In this paper, the difference in the behavior of the four vials was not negligible, and it has been taken into account as a measurement reproducibility that, in turn, affects the *K_v_* measurements.

The ending point of the primary drying stage *t_drying_* is a quantity that must be known in Equation (1a), and it has been assessed (for the sucrose solution) with regards to analyzing the curves of Figure 3A, but for the reasons already discussed, when using the temperature measurement for its definition, an uncertainty exists that will therefore affects the values of *K_v_* and, thus, of *R_p_*. It can be seen that the ice sublimation is completed about 18 h after the onset of the primary drying stage; this value can also be confirmed by the pressure ratio shown in Figure 3B, as this curve reaches the lower asymptote at the same moment. As discussed in the Section 3, this technique also allows us to identify the end of primary drying with uncertainty, but the corresponding value at the offset of the curve should correspond to the end of drying, even in the last vials of the batch.

From a measurement point of view, a better analysis of the temperature curves highlights that the ending point can be assumed as a random variable having a uniform distribution with values between a minimum of 17 h and a maximum of 19 h.

Temperature *T_B_* is recorded during the full drying process, but when the ice sublimation is approaching the end, i.e., after the time instant when the “sudden” temperature change occurs, the temperature is no longer meaningful; anyway, it is possible to assume that, in case the temperature of the heating shelf and the pressure in the drying chamber are not modified, then the slope of the temperature curve does not change till the end of the primary drying. The missing part of the temperature measures can thus just be hypothesized, and in this work it has been assumed constant until the end of the drying, the slope being very small.

#### 4.1.2. *K_v_* Estimation and Uncertainty

The values of *K_v_* obtained with Equation (1a) for three different values of assumed drying time (minimum, maximum and mean value, uniform time distribution) are shown in Table 1; the results obtained using the four temperature measurements available from the test run are compared. The table also reports the maximum deviation of *K_v_* with respect to the mean value, both for the drying time and for the monitored vials. It appears that *K_v_* is mainly affected by the drying time (±5.4%) and that the dispersion of the results obtained among the four vials is about ±1.8%, which is a small but not negligible value.

The uncertainty of *K_v_* can now be obtained by propagating the main uncertainty contributions that appear in Equation (1a) using the procedure described in the “Guide to the Expression of Uncertainty in Measurement” [54,55]. Drying time and temperature uncertainties are considered here, whereas the uncertainties related to the vial area *A_v_* and water mass *m* can be neglected. Effects related to temperature differences recorded in different vials are considered a reproducibility contribution and treated in a statistical way. Other aspects, such as the thermocouple wires that perturb the vial sublimation process and the product temperature, which can only be hypothesized towards the end of the drying, have not been considered here, since their role looks negligible after a preliminary investigation.

The approach described in [54] requires knowledge of the standard uncertainties and their sensitivity coefficients, as well as the correlation coefficients. The drying time can be assumed to be a random variable uniformly distributed between 17 h and 19 h, as said before. Thus, its expected value is 18 h, and the uncertainty can be obtained using the type-B method, that is, a method of evaluation of uncertainty not based on the statistical analysis of series of observations. The standard uncertainty is thus *u*^B^(*t_drying_*)= 1/√3 h, where the suffix B refers to a type-B method employed here.

The sensitivity coefficient of *K_v_* with respect to the drying time can be assessed from Table 1 or numerically with Equation (1a) as Δ*K_v_*/Δ*t_drying_*, thus obtaining $S_{t_{drying}}^{K_{v}}$= 1.0 Wm^−2^K^−1^/h, regardless of the considered vial.

Temperature measurements for the *K_v_* computation were obtained using two different thermometers.

The product temperature *T_B_* was measured in different vials using thermocouple sensors. A maximum error of ± 1 °C was considered, and thus, the standard uncertainty could be obtained using a type-B method considering a uniform distribution, obtaining *u*^B^(*T_B_*)=1/√3 °C.

The shelf temperature *T_shelf_* is the fluid temperature measured and controlled by the freeze-dryer. A maximum deviation of ±1 °C from the set-point *T_shelf_*= −20 °C has been considered in this uncertainty evaluation. As for *T_B_*, the standard uncertainty of *T_shelf_* is *u*^B^(*T_shelf_*)=1/√3 °C.

Moreover, temperatures in Equation (1a) are integrated during the drying phase, and the integral has been approximated as a sum of temperatures sampled every minute. These repeated measurements are considered fully correlated here because the temperature does not present large changes during the drying, and the thermometer noise can be assumed negligible with respect to the calibration uncertainty that affects all the measurements. On the other hand, temperature measurement *T_B_* can be considered fully uncorrelated with respect to the measurement *T_shelf_*, these two quantities being obtained or controlled using different thermometers.

The sensitivity of *K_v_* with respect to *T_shelf_* and *T_B_* has been obtained numerically, introducing a small temperature perturbation during the computation of Equation (1a). The two sensitivities are very similar, and they do not depend significantly on the considered vial. The obtained result is $S_{T_{B}}^{K_{v}}$ ≅ $S_{T_{shelf}}^{K_{v}}$ ≅ 1.2 W·m^−2^·K^−1^/K.

The uncertainty contribution related to the different temperature profiles recorded in the different vials (#1 to #4 in Table 1) can be estimated with a type-A method, that is, as the experimental standard deviation of the mean, obtained by processing the four different *K_v_* values when the drying time is 18 h, thus obtaining a standard uncertainty of the average *u*^A^(*vial*) = 0.16W·m^−2^·K^−1^.

Eventually, the combined standard uncertainty of $K_{v}$ is:

(8) $$u_{c}{\left(K_{v}\right)}^{2}=S_{t_{drying}}^{K_{v}}{}^{2}\cdot u^{B}{\left(t_{drying}\right)}^{2}+S_{T_{shelf}}^{K_{v}}{}^{2}\cdot u^{B}{\left(T_{shelf}\right)}^{2}+S_{T_{B}}^{K_{v}}{}^{2}\cdot u^{B}{\left(T_{B}\right)}^{2}+u^{A}{\left(vial\right)}^{2}$$

In conclusion, the measurement of *K_v_* is the mean of the four values obtained at time 18 h, and it is *K_v_*=18.7 W·m^−2^·K^−1^, with an expanded uncertainty (coverage factor 2, confidence level 95%) *U*=2.4W·m^−2^·K^−1^, which is about 13% when expressed in relative form.

This result also shows that temperature measurements represent the main uncertainty contributions and thus the thermometer accuracy, as well as the correlation analysis among measurements, deserve a deeper investigation.

The same analysis can be carried out for the polyvinylpyrrolidone solution: looking at the temperature profiles shown in Figure 3C, it appears that the trend is similar to that shown in graph A for the sucrose solution, and in this case there is also an uncertainty in the estimation of the drying time that could range between 17 and 20 h. Table 2 shows the calculated values of *K_v_,* assuming different values of the drying time. In this case, the dispersion on the estimated value of *K_v_* related to the assumption about the drying time is slightly higher than in the case of the sucrose solution (±7.8% max vs. 5.4% max).

The *K_v_* uncertainty can be estimated with the same procedure described for the test with sucrose solution. The sensitivity coefficients are almost the same, but the standard uncertainties of the drying time and reproducibility are slightly larger (from data in Table 2: *u*^B^(*t_dying_*)= 1.5/√3 h and *u*^A^(*vial*) = 0.30W·m^−2^·K^−1^ ).

The measurement of *K_v_*, computed as the mean value of the *K_v_* values obtained with the four vials at the mean drying time of 18.5 h, is *K_v_* =17.4 W·m^−2^·K^−1^, with an expanded uncertainty (coverage factor 2, confidence level 95%) *U*=2.4 W·m^−2^·K^−1^, that is, about 16% in relative form. The *K_v_* measurements with sucrose and with PVP are thus in agreement.

The heat transfer coefficient has been also measured with the gravimetric procedure for comparison purposes. To this aim, a different test was carried out in the same nominal conditions but for a shorter duration that was about half the full drying time. The mass of a large set of vials was measured using an analytical balance. Eventually, the coefficient was obtained with Equation (1b).

The uncertainty estimation follows the same procedure described for the two previous tests, but in this case:

- The drying time *t*_1_ is defined by the user, and its uncertainty is negligible.
- A set of about 100 vials was identified as representative of the full batch. The vials were weighed before and after the test, thus obtaining a set of Δ*m* measurements and, with Equation (1b), a set of *K_v_* coefficients. The mean value is 18.7 W·m^−2^·K^−1^ and the dispersion can be treated using a Type-A method, thus obtaining a standard deviation of about 2 W·m^−2^·K^−1^ and a standard uncertainty, that is, the standard deviation of the mean value, of about *u*^A^(*vial*) = 0.2 W·m^−2^·K^−1^.

As in the previous case, temperature measurements were considered fully correlated among the *T_B_* measurements and fully uncorrelated with *T_Shelf_*; the sensitivity coefficients were numerically computed from Equation (1b), thus obtaining results very similar to the ones obtained in the previous tests. Moreover, the gravimetric test was performed using the same thermometers, and thus, the temperature uncertainties are also the same, that is, *u*^B^(*T_Shelf_*) = *u*^B^(*T_B_*) = 1/√3 °C. The overall uncertainty is:

(9) $$u_{c}{\left(K_{v}\right)}^{2}=S_{T_{shelf}}^{K_{v}}{}^{2}\cdot u^{B}{\left(T_{shelf}\right)}^{2}+S_{T_{B}}^{K_{v}}{}^{2}\cdot u^{B}{\left(T_{B}\right)}^{2}+u^{A}{\left(vial\right)}^{2}$$

The heat transfer coefficient *K_v_* measured with the gravimetric test is thus *K_v_* = 18.7 W·m^−2^·K^−1^ with an expanded uncertainty (coverage factor 2, confidence level 95%) *U* = 2 W·m^−2^·K^−1^, about 11% in relative form.

**Figure 4:**
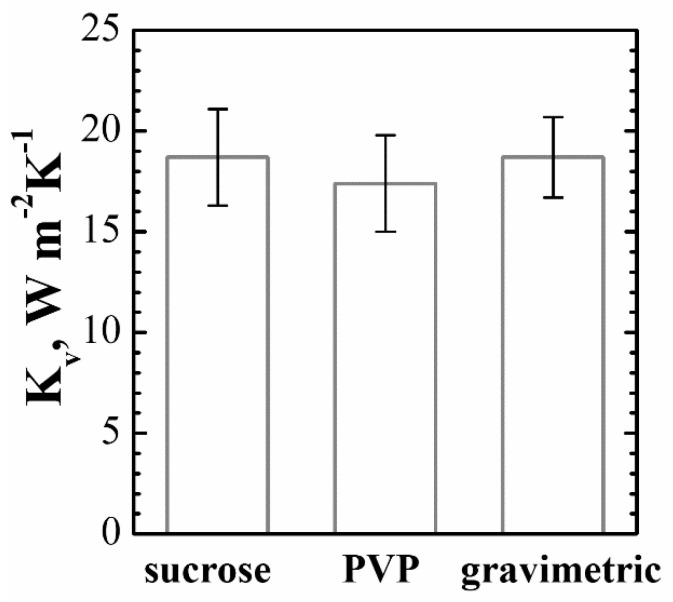
Values of *K_v_* obtained from the measurements in the run with the sucrose solution, in the run with the PVP solution, and in the run where a gravimetric test was carried out. The intervals represent the expanded uncertainty with coverage factor 2. The contributions considered here are the drying time and the temperature measurements, as well as the dispersion among vials.

Figure 4 shows the *K_v_* measures obtained from recording the full drying of sucrose and PVP, as well as the measurement result from the gravimetric test. The results are in good agreement, with a small difference in the measured values. One should note that the proposed method has a larger uncertainty but also the advantage of saving time, and it can be carried out during the manufacturing run.

#### 4.1.3. *Rp* Estimation and Monitoring of Primary Drying Progress

**Figure 5:**
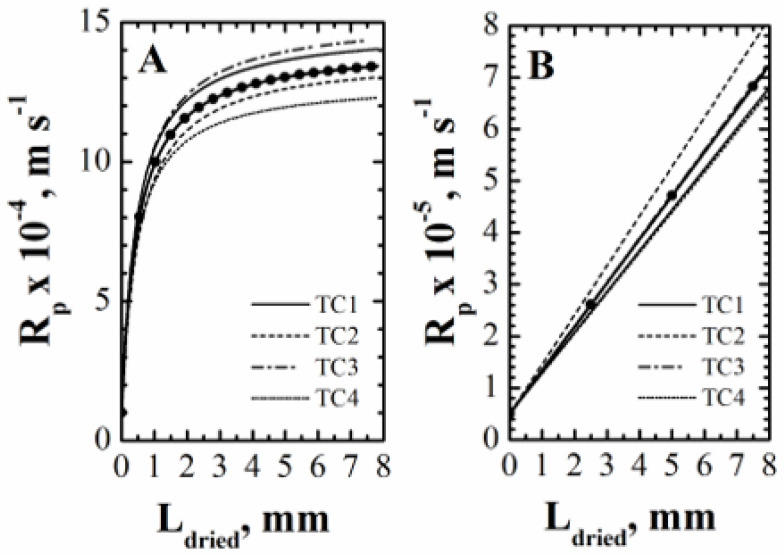
*R_p_* vs. *L_dried_* calculated using the various temperature measurements (lines) and the calculated mean values (symbols) of the parameters expressing the dependence of *R_p_* on *L_dried_* for the sucrose solution (graph **A**) and the PVP solution (graph **B**).

Once the value of *K_v_* has been estimated, the curve of *R_p_* vs. *L_dried_* can be obtained, and then the three values of the parameters *R_p_*_,0_, $A_{R_{p}}$, and $B_{R_{p}}$ can be calculated. According to the algorithm previously described, the measurement of product temperature is required to obtain the curve of *R_p_* vs. *L_dried_*, and thus, as various temperature measurements are usually available, different curves of *R_p_* vs. *L_dried_* are obtained, i.e., different sets of values of *R_p_*_,0_, $A_{R_{p}}$, and $B_{R_{p}}$ (see Figure 5). The approach used in this study is the following:

- estimate the values of *R_p_*_,0_, $B_{R_{p}}$, and $A_{R_{p}}$ from the curve of *R_p_* vs. *L_dried_* obtained from the first temperature measurement;
- use the previously obtained values of *R_p_*_,0_ and $B_{R_{p}}$ to calculate the value of $A_{R_{p}}$ in such a way that the data of *R_p_* vs. *L_dried_* obtained from the other temperature measurements can be best-fitted;
- calculate the mean value of $A_{R_{p}}$ and its standard deviation.

**Figure 6:**
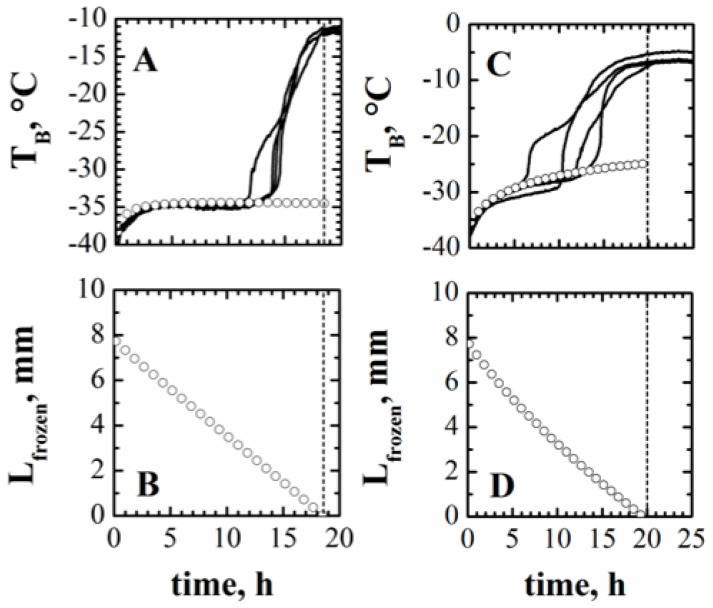
Comparison between measured (lines) and calculated (symbols) values of product temperature (**A**,**C**) and calculated evolution of the thickness of the frozen product (**B**,**D**) for the freeze-drying of the 5% by weight sucrose solution (**A**,**B**) and of the 5% by weight PVP solution (**C**,**D**). Dashed lines identify the ending point of the primary drying stage.

For the first product, considering the four different temperature measurements and the four different *R_p_* vs. *L_dried_* curves it is possible to obtain (mean value ± standard deviation): *R_p,_*_0_ = 10^4^ m·s^−1^, $A_{R_{p}}$= 2.84·10^8^ s^−1^ ± 7.6%, and $B_{R_{p}}$ = 2.16·10^3^ m^−1^. Similarly, for the second product, it is possible to obtain *R_p,_*_0_ = 5·10^4^ m·s^−1^, $A_{R_{p}}$ = 8.43·10^7^ s^−1^ ± 9.61%, and $B_{R_{p}}$= 0 m^−1^. The standard deviation on $A_{R_{p}}$ is in agreement with that reported in the literature when other methods were used to estimate it [48]. With respect to the estimated *R_p_* values, it is impossible to compare them with a value measured using a different technique, as it was performed for the coefficient *K_v_*. Nevertheless, it is possible to evaluate whether these estimates are correct by running a simulation using the mathematical model of the process with the estimated values of model parameters, then comparing calculated and measured values of product temperature and process duration. Results are shown in Figure 6 for both products, evidencing the accuracy of both the temperature values calculated and the drying time (i.e., the time when *L_frozen_* becomes equal to zero). Graphs B and D of Figure 6 also evidence that even if the direct measurement of the interface position is not possible in normal process conditions, using the proposed PAT and Equation (5), by means of an inferential approach, it may be possible to also monitor this variable, and thus the progress of the primary drying.

### 4.2. Strengths and Weaknesses of Thermocuples for Pharmaceutical Applications in Comparison with a Contactless Device (IR Camera)

The previous section demonstrated that thermocouples may become a reliable and efficient PAT tool which allows us to monitor the process and estimate the relevant parameters with an uncertainty comparable to more time-consuming approaches. Attention must be paid, anyway, to the fact that when using thermocouples or other contact measuring devices, the measured value is strongly influenced by the close environment of the sensing element and by the quality of contact with the product, as discussed by Bosca et al. [33]; this may be responsible for either anticipated temperature rise during primary drying, a consequence of preferential drying or microcollapse along the thermocouple wire, or slower temperature increase for the presence of residual ice.

What makes thermocouples widely employed in the pharmaceutical field, especially at pilot scale and in the development step, is the facility of use and possibility to easily place them in various positions all over the apparatus, thus allowing us to monitor the core and edge zones over different shelves. In fact, the contribution of the different heat transfer mechanisms changes with the position of the product on the shelf: the product at the edges of the shelf may also be heated by radiation from the chamber walls; core and border vials may have a different contribution by conduction. This leads to differences in the process parameters, not only in *K_v_* but also in *R_p_*, because the different freezing conditions may lead to differences in the ice structure and then in the cake porosity, which will be reflected in differences in product temperature and the residual amount of water from vial to vial. Placement of thermocouples in certain zones of a large apparatus may be limited by accessibility, but the use of a system with wireless signal transmission (two different solutions have been presented, for example, in [38,39]) may help solve the problem; at the moment, anyway, contact devices are the only ones that may allow a complete mapping, and the use of wireless modules allows us to increase, with virtually no limitations, the number of probes utilizable without any modifications of the apparatus.

On the other hand, wired measurement systems require a dedicated vacuum stopper and, when the thermocouple signals are measured outside the chamber, a more complex calibration procedure [56].

Contactless devices such as IR cameras, in this respect, have many more limitations. If placed outside the chamber on the top of the freeze-dryer, the sensor, through a germanium window, was able to measure the product temperature on the top surface of the upper shelf, the only one that the camera could see [40]. In order to also measure the whole axial temperature profile, Emteborg et al. [41] used custom-made cuvettes with a germanium window on one side, placed close to an IR mirror with a 45° angle in such a way that the IR radiation from the cuvette was reflected upwards to the IR camera. It is evident that such a system requires strong hardware modifications and is not widely applicable.

The use of an IR camera inside the chamber allows for much more flexibility and the possibility of using it in an existing device with no modifications, if a passthrough for connections is available. Obviously, only the shelf with the camera and only vials at the edge of the shelf may be monitored. This may be a strong limitation because, as discussed before, the edge vials generally receive more heat and are not representative of the core of the batch. Harguindeguy and Fissore [45] showed that, taking advantage of the hexagonal arrangement of the vials in the batch, it is possible to acquire temperature measurements for both first- and second-row vials from the image obtained, which might be considered representative of “edge” and (with sufficient approximation) “central” vials.

Compared to thermocouples, which have limited measurement and calibration issues, the use of an infrared camera inserted in the drying chamber presents several problems. The first is the accuracy of the temperature measurement: in particular, the emissivity of the object has to be detected—for example, by using the ISO 18434-136 guideline [57] (Part 1, Annex A.2, E) as well as the “reflected apparent temperature” due to the radiations emitted by the surrounding environment at the measured object, which reflects the same at the camera with its radiation. The second issue is the object detection and segmentation, as it is necessary to identify and segment the objects whose temperatures have to be measured and to track them in case of movement due to equipment vibrations, correlating information across subsequent images [44]. Thus, the data acquired require a complex post-processing; the calibration is generally performed via comparison with another temperature measurement with a final higher uncertainty and with a spatial resolution related to the pixel size of the image.

On the other hand, contactless devices obviously do not interfere directly with the process, apart from possible radiation effects. The unique capacity of the sensor, which is placed in the drying chamber, to track the whole axial temperature profile can be exploited in the freezing stage to estimate the mean size of the ice crystals and, from this, the resistance of the dried cake to vapor flux (*R_p_*), as the size of the pores corresponds to that of the ice crystals (in case collapse does not occur) [58]. Recent work by Harguindeguy et al. [59] has also shown how the infrared camera may be effective in investigating the freezing phenomena when controlled nucleation is used (e.g., vacuum-induced surface freezing) and when non-conventional vial loading is used.

On the contrary, the thermocouple inserted in the vial might interfere, especially in case of liquid products. In fact, its presence might favor the nucleation of ice crystals, altering the final cake structure, or might modify heat transfer mechanisms, acting as a fin. It was evidenced by Bosca et al. [33] that in non-GMP conditions, which are those generally encountered in laboratory and development stages, the effect of this interference may be neglected, but it may be relevant in the freezing step in a production environment and should be further investigated. The problem can be solved using thermistors or thermocouples sputtered on the external of the vial: such a system has also been proven to be effective for monitoring processes with controlled nucleation [36]. In this case, the vial itself becomes a PAT tool and must be prepared on purpose, but any commercial vial can be employed, and it is also possible to obtain a thermocouple array with several measuring points [35]. Of course, in this case the temperature of the glass vial is measured (as in the case of using the IR camera). It has been shown via modeling that the difference between the product and the vial may be very small [47], but it must be remembered that this is true only if there is good contact between the product and the vial; this is generally true for the frozen product, while the dried cake may shrink, detaching from the wall.

When comparing contact and contactless sensors, it must be considered that in the first case, in general, only one punctual measurement is available (even if devices with multiple measuring point have been proposed), and the size of the measuring element and its correct position are critical. With thermocouples, the sensing tip may be very tiny, but its real location can be different from the assumed one as a consequence of deformations, undesired movements, and human errors [60]. Infrared cameras allow us to measure the whole temperature profile along the axis of the vial, but the measurements are affected by the accuracy of the sensor, and the resolution is related to the pixel size.

To complete the comparison between contact and contactless devices, their use for process monitoring and optimization must be considered. Using thermocouples, the values of model parameters can be obtained only at the end of the primary drying stage, and thus the system can be used only for off-line optimization and not for real-time control and optimization, unless a different algorithm is used—for example, using a robust soft-sensor [46,61]. Obviously, once the parameters have been determined, the PAT tool can be used to monitor the progress of the drying in-line, estimating the position of the frozen interface, as shown.

Similarly, when the infrared camera is used, the model parameters *K_v_* and *R_p_* may be estimated at the end of the primary drying [43] and used to calculate the design space of the primary drying step [62], as well as in-line, taking advantage of the detection of the moving sublimation interface (where the temperature of the product is lower) and, thus, of the thickness of the frozen layer [45,62]. Furthermore, the temperature detected may be used to identify the ending point of the primary drying step, together with the estimated thickness of the frozen layer [45], even if the measurement may be quite noisy.

## 5. Conclusions

Temperature sensors based on different technologies, coupled with a model, can become a powerful PAT tool. They can be used to monitor freeze-drying processes and to estimate both the model parameters to be used for off-line process optimization and the minimum primary drying time, thus avoiding prolonging this stage unnecessarily. The described procedure can, in principle, be applied to both contact and contactless sensors, even if sources of error and uncertainty are different in the two cases. In this paper, a tool based on thermocouples inserted in the vial was developed and tested, analyzing the main uncertainty contributions from a metrological point of view. It was shown that the variables and parameters of interest can be estimated with an accuracy similar to that obtained using other measuring systems, but the hardware involved is less expensive, and it is not required to carry out any preliminary investigation to obtain the values of model parameters since they can be retrieved during a manufacturing run, thus saving time and money.

The PAT tool was developed for the configuration of general interest in pharmaceutical manufacturing, with heat supplied only through the shelf and the bottom of the container; in this case, the bottom temperature *T_B_*, which is the measured variable, is the highest and thus the most relevant temperature, but the interface temperature may also be estimated, which is useful for a more accurate evaluation of mass transfer in the cake. Modifications will be necessary to adapt the system to be employed if the heat flow from the chamber walls and the upper shelf is significant, because the temperature profiles in the product will be affected.

Thermocouples are the most employed temperature sensors since they represent an excellent trade-off among cost, accuracy, invasiveness, ease of use, and sensor interchangeability. Anyway, the effect on the drying process is still an open issue, since these sensors are in contact with the product. They can both affect the nucleation process locally, because they introduce a local discontinuity, and speed up the drying process, for example, because the sensor wires introduce a heat path from the vial to the product. For these reasons, contactless devices were proposed, and their performances were compared here to those of the thermocouples, evidencing reciprocal strengths and weaknesses. The infrared camera may be able to solve several issues with respect to contact probes, as the absence of any contact between the sensor and the product undergoing freeze-drying prevents any effect on ice nucleation. The tool may surely be useful in the stage of process development carried out at lab scale, where it can be manually inserted in the equipment, monitoring the dynamics of the vials of interest. They are effective but still not so easy to use, and commercial sensors based on these approaches are not yet available. Application at an industrial (manufacturing) scale is more tricky due to the automatic systems for loading/unloading vials, unless it is directly embedded in the drying chamber.

## Figures and Tables

**Table 1:** Values of *K_v_* (W m^−2^ K^−1^) for sucrose solution estimated from different drying times and considering the temperature measured in the 4 vials.

*t_drying_* (h)	Sucrose—Vial				$\Delta K_{v}$ $\left(\Delta K_{v}/\bar{K_{v}}\right)$
	#1	#2	#3	#4	
17	20.07	19.65	20.17	19.45	±0.36 (±1.8%)
18	18.95	18.56	19.04	18.35	±0.35 (±1.9%)
19	18.04	17.66	18.13	17.82	±0.34 (±1.9%)
$\Delta K_{v}$ ($\Delta K_{v}/\bar{K_{v}}$)	±1.0 (±5.4%)	±1.0 (±5.4%)	±1.0 (±5.3%)	±1.0 (±5.4%)	

**Table 2:** Values of *K_v_* (W·m^−2^·K^−1^) for PVP solution estimated from different drying times and considering the temperature measured in the 4 vials.

*t_drying_* (h)	PVP—Vial				$\Delta K_{v}$ $\left(\Delta K_{v}/\bar{K_{v}}\right)$
	#1	#2	#3	#4	
17	19.34	18.69	17.93	19.13	±0.71 (±3.8%)
18.5	17.91	17.26	16.57	17.71	±0.67 (±3.9%)
20	16.64	15.98	15.36	16.44	±0.64 (±4.0%)
$\Delta K_{v}$ ($\Delta K_{v}/\bar{K_{v}}$)	±1.4 (±7.5%)	±1.4 (±7.8%)	±1.3 (±7.8%)	±1.3 (±7.6%)	

## Data Availability

Data are contained within the article.

## Symbols

$A_{R_{p}}$	fitting parameter for cake resistance relationship in Equation (7)
*A_v_*	cross-section area of the vial
$B_{R_{p}}$	fitting parameter for cake resistance relationship in Equation (7)
∆*H_s_*	heat of ice sublimation
*J_q_*	heat flux to the product
*J_w_*	sublimation flux
*k_frozen_*	thermal conductivity of the frozen layer
*K_v_*	heat transfer coefficient
*L*	thickness of the product
*L_dried_*	thickness of the dried product
*L_froze_*_n_	thickness of the frozen layer
*m*	mass of ice in the vial
∆*m*	variation of ice by sublimation in the test
*p_w,i_*	water vapor partial pressure at the interface of sublimation
*p_w,c_*	water vapor partial pressure in the drying chamber
*R_p_*	resistance to mass transfer
*R_p,_*_0_	fitting parameter for cake resistance relationship (7)
$S_{x}^{y}$	sensitivity coefficient of the output quantity *y* with respect to the input quantity *x* evaluated at the measurement values
*T_B_*	product temperature at the bottom of the vial
*T_i_*	product temperature at the interface of sublimation
*T_shelf_*	shelf (or fluid) temperature
*t*	time
*t_drying_*	time required to complete the ice sublimation
*U*	expanded uncertainty
*u* ^A^ *(x)*	standard uncertainty of the quantity *x* evaluated with type-A method
*u* ^B^ *(x)*	standard uncertainty of the quantity *x* evaluated with type-B method
*u*_c_ *(x)*	combined uncertainty of the quantity *x*
*v_i_*	interface retreating velocity
*ρ_dried_*	density of the dried product
*ρ_frozen_*	density of the frozen product

## References

[1] Jennings T.A. (1999). Lyophilization: Introduction and Basic Principles.

[2] Oetjen G.W., Haseley P. (2004). Freeze-Drying.

[3] Franks F., Auffret T. (2007). Freeze-Drying of Pharmaceuticals and Biopharmaceuticals.

[4] Fissore D. (2013). Freeze Drying of Pharmaceuticals. Encyclopedia of Pharmaceutical Science and Technology.

[5] Bellows R.J., King C. (1972). Freeze-drying of aqueous solutions: Maximum allowable operating temperature. Cryobiology.

[6] Pikal M.J., Cleland J.L., Langer R. (1994). Freeze-drying of proteins: Process, formulation, and stability. Formulation and Delivery of Proteins and Peptides.

[7] Franks F. (1998). Freeze-drying of bioproducts: Putting principles into practice. Eur. J. Pharm. Biopharm..

[8] Chen Y., Mutukuri T.T., Wilson N.E., Zhou Q. (2021). Pharmaceutical protein solids: Drying technology, solid-state characterization and stability. Adv. Drug Deliv. Rev..

[9] Wang B., Pikal M.J. (2012). Stabilization of lyophilized pharmaceuticals by process optimization: Challenges and opportunities. Am. Pharm. Rev..

[10] Tsourouflis S., Flink J.M., Karel M. (1976). Loss of structure in freeze-dried carbohydrates solutions: Effect of temperature, moisture content and composition. J. Sci. Food Agric..

[11] Depaz R.A., Pansare S., Patel S.M. (2016). Freeze-drying above the glass transition temperature in amorphous protein formulations while maintaining product quality and improving process efficiency. J. Pharm. Sci..

[12] Patel S.M., Nail S.L., Pikal M.J., Geidobler R., Winter G., Hawe A., Davagnino J., Gupta S.R. (2017). Lyophilized drug product cake appearance: What is acceptable?. J. Pharm. Sci..

[13] Johnson R., Lewis L. (2011). Freeze-drying protein formulations above their collapse temperatures: Possible issues and concerns. Am. Pharm. Rev..

[14] Barresi A.A., Fissore D. (2011). In-line product quality control of pharmaceuticals. Freeze-Drying Processes.

[15] Fissore D., Pisano R., Barresi A.A. (2015). Using mathematical modeling and prior knowledge for QbD. Freeze-Drying Processes.

[16] Food and Drug Administration (2004). Guidance for Industry PAT—A Framework for Innovative Pharmaceutical Manufacturing and Quality Assurance. https://www.fda.gov/downloads/drugs/guidances/ucm070305.pdf.

[17] Barresi A., Pisano R., Fissore D., Rasetto V., Velardi S.A., Vallan A., Parvis M., Galan M. (2009). Monitoring of the primary drying of a lyophilization process in vials. Chem. Eng. Process. Process. Intensif..

[18] Nail S., Tchessalov S., Shalaev E., Ganguly A., Renzi E., Dimarco F., Wegiel L., Ferris S., Kessler W., Pikal M. (2017). Recommended best practices for process monitoring instrumentation in pharmaceutical freeze drying—2017. AAPS PharmSciTech.

[19] Fissore D., Pisano R., Barresi A.A. (2018). Process analytical technology for monitoring pharmaceuticals freeze-drying—A comprehensive review. Dry. Technol..

[20] Patel S.M., Doen T., Pikal M.J. (2010). Determination of end point of primary drying in freeze-drying process control. AAPS PharmSciTech.

[21] Pisano R. (2022). Automatic control of a freeze-drying process: Detection of the end point of primary drying. Dry. Technol..

[22] Milton N., Pikal M.J., Roy M.L., Nail S.L. (1997). Evaluation of manometric temperature measurement as a method of monitoring product temperature during lyophilization. PDA J. Pharm. Sci. Technol..

[23] Chouvenc P., Vessot S., Andrieu J., Vacus P. (2004). Optimization of the freeze-drying cycle: A new model for pressure rise analysis. Dry. Technol..

[24] Velardi S.A., Rasetto V., Barresi A.A. (2008). Dynamic Parameters Estimation method: Advanced Manometric Temperature Measurement approach for freeze-drying monitoring of pharmaceutical solutions. Ind. Eng. Chem. Res..

[25] Fissore D., Pisano R., Barresi A. (2011). On the methods based on the Pressure Rise Test for monitoring a freeze-drying process. Dry. Technol..

[26] Pisano R., Ferri G., Fissore D., Barresi A.A. Freeze-drying monitoring via Pressure Rise Test: The role of the pressure sensor dynamics. Proceedings of the Instrumentation and Measurement Technology Conference—IMTC 2017.

[27] Schneid S.C., Gieseler H., Kessler W.J., Pikal M.J. (2009). Non-invasive product temperature determination during primary drying using Tunable Diode Laser Absorption Spectroscopy. J. Pharm. Sci..

[28] Rasetto V., Marchisio D.L., Fissore D., Barresi A.A. (2010). On the use of a dual-scale model to improve understanding of a pharmaceutical freeze-drying process. J. Pharm. Sci..

[29] Barresi A.A., Pisano R., Fissore D., Martynenko A., Bück A. (2018). Advanced control in freeze-drying. Intelligent Control in Drying.

[30] Vollrath I., Pauli V., Friess W., Freitag A., Hawe A., Winter G. (2017). Evaluation of heat flux measurement as a new process analytical technology monitoring tool in freeze drying. J. Pharm. Sci..

[31] Schneid S., Gieseler H. (2008). Evaluation of a new wireless Temperature Remote Interrogation System (TEMPRIS) to measure product temperature during freeze drying. AAPS PharmSciTech.

[32] Kasper J.C., Wiggenhorn M., Resch M., Friess W. (2013). Implementation and evaluation of an optical fiber system as novel process monitoring tool during lyophilization. Eur. J. Pharm. Biopharm..

[33] Bosca S., Barresi A.A., Fissore D. (2013). Use of a soft sensor for the fast estimation of dried cake resistance during a freeze-drying cycle. Int. J. Pharm..

[34] Grassini S., Parvis M., Barresi A.A. (2013). Inert thermocouple with nanometric thickness for lyophilization monitoring. IEEE Trans. Instrum. Meas..

[35] Parvis M., Grassini S., Fulginiti D., Pisano R., Barresi A.A. Sputtered thermocouple array for vial temperature mapping. Proceedings of the 2014 IEEE International Instrumentation and Measurement Technology Conference (I2MTC) Proceedings.

[36] Oddone I., Fulginiti D., Barresi A.A., Grassini S., Pisano R. (2015). Non-invasive temperature monitoring in freeze drying: Control of freezing as a case study. Dry. Technol..

[37] Jiang X., Kazarin P., Sinanis M.D., Darwish A., Raghunathan N., Alexeenko A., Peroulis D. (2022). A non-invasive multipoint product temperature measurement for pharmaceutical lyophilization. Sci. Rep..

[38] Corbellini S., Parvis M., Vallan A. (2010). In-process temperature mapping system for industrial freeze dryers. IEEE Trans. Instrum. Meas..

[39] Bosca S., Corbellini S., Barresi A.A., Fissore D. (2013). Freeze-drying monitoring using a new Process Analytical Technology: Toward a “zero defect” process. Dry. Technol..

[40] Emteborg H., Zeleny R., Charoud-Got J., Martos G., Lüddeke J., Schellin H., Teipel K. (2014). Infrared thermography for monitoring of freeze-drying processes: Instrumental developments and preliminary results. J. Pharm. Sci..

[41] Emteborg H., Charoud-Got J., Seghers J. (2022). Infrared thermography for monitoring of freeze drying processes—Part 2: Monitoring of temperature on the surface and vertically in cuvettes during freeze drying of a pharmaceutical formulation. Pharmaceutics.

[42] Van Bockstal P.-J., Corver J., De Meyer L., Vervaet C., De Beer T. (2018). Thermal imaging as a noncontact inline Process Analytical Tool for product temperature monitoring during continuous freeze-drying of unit doses. Anal. Chem..

[43] Lietta E., Colucci D., Distefano G., Fissore D. (2019). On the use of infrared thermography for monitoring a vial freeze-drying process. J. Pharm. Sci..

[44] Colucci D., Morra L., Zhang X., Fissore D., Lamberti F. (2020). An automatic computer vision pipeline for the in-line monitoring of freeze-drying processes. Comput. Ind..

[45] Harguindeguy M., Fissore D. (2021). Temperature/end point monitoring and modelling of a batch freeze-drying process using an infrared camera. Eur. J. Pharm. Biopharm..

[46] Fissore D., Pisano R., Barresi A.A. On the use of temperature measurement to monitor a freeze-drying process for pharmaceuticals. Proceedings of the 2017 IEEE International Instrumentation and Measurement Technology Conference (I2MTC).

[47] Velardi S.A., Barresi A.A. (2008). Development of simplified models for the freeze-drying process and investigation of the optimal operating conditions. Chem. Eng. Res. Des..

[48] Giordano A., Barresi A.A., Fissore D. (2011). On the use of mathematical models to build the design space for the primary drying phase of a pharmaceutical lyophilization process. J. Pharm. Sci..

[49] Fissore D., Pisano R., Barresi A.A. (2011). Advanced approach to build the design space for the primary drying of a pharmaceutical freeze-drying process. J. Pharm. Sci..

[50] Pisano R., Fissore D., Velardi S.A., Barresi A. (2010). In-line optimization and control of an industrial freeze-drying process for pharmaceuticals. J. Pharm. Sci..

[51] Fissore D., Pisano R., Barresi A.A. (2012). Model-based framework for the analysis of failure consequences in a freeze-drying process. Ind. Eng. Chem. Res..

[52] Oddone I., Van Bockstal P.-J., De Beer T., Pisano R. (2016). Impact of vacuum-induced surface freezing on inter- and intra-vial heterogeneity. Eur. J. Pharm. Biopharm..

[53] Capozzi L.C., Pisano R. (2018). Looking inside the ‘black box’: Freezing engineering to ensure the quality of freeze-dried biopharmaceuticals. Eur. J. Pharm. Biopharm..

[54] BIPM, IEC, IFCC, ILAC, ISO, IUPAC, IUPAP, OIML. Evaluation of Measurement Data – Guide to the Expression of Uncertainty in Measurement, JCGM 100:2008. https://www.bipm.org/documents/20126/2071204/JCGM_100_2008_E.pdf/cb0ef43f-baa5-11cf-3f85-4dcd86f77bd6.

[55] Uncertainty of Measurement—Part 3: Guide to the Expression of Uncertainty in Measurement (GUM:1995).

[56] (2021). Standard Guide for Thermocouple Verification.

[57] Condition Monitoring and Diagnostics of Machines—Thermography—Part 1: General Procedures.

[58] Colucci D., Maniaci R., Fissore D. (2019). Monitoring of the freezing stage in a freeze-drying process using IR thermography. Int. J. Pharm..

[59] Harguindeguy M., Stratta L., Fissore D., Pisano R. (2021). Investigation of the freezing phenomenon in vials using an infrared camera. Pharmaceutics.

[60] Demichela M., Barresi A.A., Baldissone G. (2018). The effect of human error on the temperature monitoring and control of freeze drying processes by means of thermocouples. Front. Chem..

[61] Bosca S., Barresi A.A., Fissore D. (2015). Design of a robust soft-sensor to monitor in-line a freeze-drying process. Dry. Technol..

[62] Harguindeguy M., Fissore D. (2021). Micro freeze-dryer and infrared-based PAT: Novel tools for primary drying design space determination of freeze-drying processes. Pharm. Res..

